# Extirpation of the Spleen

**Published:** 1879-05

**Authors:** Henry W. Rogers

**Affiliations:** Private student


*ART. III.—Extirpation of the Spleen.* Reported by HENRY W. ROGERS (private student).

Mrs. C., æt 40 years, first admitted to Sisters of Charity Hospital, Dec. 1878, under the care of Prof. T. F. Rochester, with a distinct hard tumor of the left side in the normal vicinity of the spleen. It had thus far passed her medical advisors under a variety of names, kidney fibroid tumor, cancer, &c. &c. Prof. R. after careful examination, pronounced it enlarged spleen. After explaining the nature and incurability of the malady, the patient was discharged to return home, her distresses not being wholly unendurable.

March 20th, 1879, was again admitted to Hospital Sisters of Charity. The tumor had now greatly increased in size, extending from ensiform cartilage to below the pubic bone, occupying the whole anterior portion of the abdominal cavity, somewhat obscuring the diagnosis, but Prof. Rochester remembering the case as found on first admission, was confident in the accuracy of his first diagnosis, and as flesh and strength were rapidly diminishing, and no hope of recovery was entertained, he suggested *extirpation of the spleen*, and invited Prof. J. F. Miner to make the operation, which was accepted with the view that nothing could be lost and *possibly* relief might be temporarily obtained.

March 24th, 1879, Dr. Miner with Prof. Rochester and his private assistants, in presence of a large number of the physicians of the city, attracted by the rarity of the operation, made incision upon the tumor in the median line of the abdomen, the incision reaching from two inches above the os pubis to one inch above the umbilicus. On reaching the spleen it easily turned out, there being no adhesions. A distinct pedicle was found, composed of the vessels and suspensory ligaments of the spleen, considerably elongated from the constant traction by weight of the tumor. This was secured by a double ligature and separated from the tumor by careful traction, thus adding somewhat to the length of the pedicle, which was finally secured in the upper angle of the wound, as is common in the treatment of the pedicle after ovariotomy. The abdomen having been sponged out with carbolized water, the wound was closed with interrupted sutures. The operation occupied about fifteen minutes, no blood being lost except that which escaped from the spleen after its removal. The patient was then placed in bed in apparently comfortable condition, and $\frac{1}{8}$ gr. morphine administered hypodermically.

At 8 o'clock in the evening, pulse 110 and very weak, skin cold, and constant vomiting produced continual commotion of the abdominal viscera, and discharge of a little blood from the pedicle or margins of the incision, and bloody serum from abdominal cavity.

Death occurred eighteen hours after the operation from exhaustion.

The spleen weighed after removal, 7 lbs. 4 ounces.

So far as we are able to learn from the annals of Medical Literature, this is the first operation for removal of the spleen ever performed in this country, although forty-one cases are on record, as having occurred in the practice of foreign Surgeons; twenty-one of this number were operated upon for chronic enlargement and Leucocythemia, of these only seven cases resulted favorably, while the remainder of the forty-one were operated upon for traumatic causes, and all terminated successfully.

In conclusion we may best quote the following paragraph from Ashurst, in which he says:

While excision would appear to be justifiable in a case of wound with protrusion of the organ, splenotomy for diseased conditions of the Viscus has not been sufficiently successful to encourage a repetition of the procedure. Hemorrhage, during or subsequent to the operation appears to have been the cause of death in most of the fatal cases.

